# Reply to the Letter to the Editor regarding the Article “Effects of Intra-articular Bone Marrow Aspirate Infiltration in the Treatment of Knee Osteoarthritis: A Clinical Study Comparing BMA versus Corticosteroid and Genicular Block.” *Rev Bras Ortop* 2025;60(2):s00451809512. DOI: 10.1055/s-0045-1809512

**DOI:** 10.1055/s-0046-1817131

**Published:** 2026-03-19

**Authors:** Renata Clazzer, Dilamar Moreira Pinto, Mariana Valois de Aquino Krause, Tale Lucas Vieira Rolim, Ricardo Lyra de Oliveira, Diego Ariel de Lima

**Affiliations:** 1 Department of Orthopedics and Traumatology, Hospital Otavio de Freitas, Recife, PE, Brazil; 2 Department of Health Sciences, Centro de Ciências Biológicas e da Saúde, Universidade Federal Rural do Semi-Árido, Mossoró, RN, Brazil

Dear Editor, Dr. Geraldo Rocha Motta Filho,

We would like to thank the authors of the letter regarding our article published in the *Brazilian Journal of Orthopedics* 1 for their observations, which we consider relevant and constructive. Scientific debate, when conducted with respect and technical grounding, is essential for the maturation of orthobiology as a field of research and clinical practice. We fully agree that the interpretation of results should be careful and that the pursuit of scientific rigor and conceptual clarity is fundamental. We therefore value the collaborative spirit of this debate, which will certainly contribute to the responsible advancement of orthobiological therapies in the management of knee osteoarthritis.

## Answers to the Three Issues Addressed

1. Methodological Aspects
  We acknowledge the limitations mentioned, including the loss to follow-up of 15 patients, which reduced the statistical power. This limitation was clearly described in the article.
The initial number (25 per group) was based on similar studies. 2 Considering an expected difference of 20% in the Western Ontario and McMaster Universities Arthritis Index (WOMAC) and an Alpha value of 5%, the sample size calculation would indicate approximately 25 to 35 patients per group to maintain 80% of power. **We recognize that a larger sample would be desirable to achieve the initially planned power, estimating potential losses to follow-up and the replacement of lost cases.** However, we chose not to expand the inclusion due to logistical and financial constraints, and we described this limitation honestly and explicitly.
2. Conceptual Confusion about the Intervention
We appreciate the observation regarding the use of dexamethasone. We clarify that the only goal of its inclusion was to standardize the clinical protocol previously adopted in our institution. The control group received the same protocol. The intra-articular dose used (≈ 0.8 mg) was lower than the usual range in which cellular toxicity has been reported (2–4 mg). 3 Furthermore, dexamethasone is a short-acting corticosteroid, with a plasma half-life of 3 to 5 hours and a biological duration of up to 72 hours, which minimizes the risk of prolonged exposure of mesenchymal stem cells (MSCs), differing from depot corticosteroids, such as triamcinolone and betamethasone, which are slow-release and have a greater cytotoxic potential.
There are in-vitro reports that high glucocorticoid levels and/or prolonged exposure to these drugs reduce the viability and chondrogenic differentiation of MSCs. However, these effects are dose- and time-dependent and do not represent a universal consensus on toxicity. Conversely, there is experimental evidence that dexamethasone at low doses stimulates chondrogenesis and potentiates transforming growth factor Beta (TGF-β), as described in the article cited by the authors of the letter to the editor (Csaki et al., 4 2008):
  “Dexamethasone, a synthetic glucocorticoid, stimulates chondrogenesis directly via the glucocorticoid receptor […]. Interestingly, dexamethasone potentiates the chondrogenic stimulation of TGF-β.”
Csaki et al. 4 do not report any deleterious effects or toxic doses, as these authors describe precisely the modulating and prochondrogenic dose-dependent role of dexamethasone.
  We acknowledge the controversies surrounding the combination of dexamethasone and bone marrow aspirate (BMA). Nevertheless, we chose to retain dexamethasone in the protocol to preserve similarity between the groups, as its absence could create an additional uncontrolled variable; as such, BMA was the only difference.
3. Interpretation of Results
  We appreciate the observation regarding the interpretation of the results. Indeed, as correctly pointed out, our findings only demonstrated a significant difference in the pain domain of the WOMAC at 6 months, with no difference in stiffness or function. This limitation was acknowledged in the discussion and conclusion of the article.
Regarding the publication on the Instagram page of the Brazilian Society of Othopedics and Traumatology (Sociedade Brasileira de Ortopedia e Traumatologia, SBOT, in Portuguese; @sbotnacional; https://www.instagram.com/p/DOonWhxE20A/?igsh=MTltcW1xaWszcXA1eA== ), we acknowledge that the post simplified the findings, and that the statement “improvement in pain and function” can be interpreted more broadly than the original scientific text. We agree that the statement could be more precise; however, we understand that it is a brief communication intended to encourage readers to consult the full article in the *Brazilian Journal of Orthopedics* .
